# Use of Wearable Activity-Monitoring Technologies to Promote Physical Activity in Cancer Survivors: Challenges and Opportunities for Improved Cancer Care

**DOI:** 10.3390/ijerph20064784

**Published:** 2023-03-08

**Authors:** Melanie R. Keats, Xing Yu, Molly Sweeney Magee, Cynthia C. Forbes, Scott A. Grandy, Ellen Sweeney, Trevor J. B. Dummer

**Affiliations:** 1 School of Health and Human Performance, Dalhousie University, Halifax, NS B3H 4R2, Canada; 2 Division of Medical Oncology, Department of Medicine, Dalhousie University & Nova Scotia Health, Halifax, NS B3H 4R2, Canada; 3 Beatrice Hunter Cancer Research Institute, Halifax, NS B3H 4R2, Canada; 4 School of Population and Public Health, University of British Columbia, Vancouver, BC V6T 1Z3, Canada; 5 Wolfson Palliative Care Research Centre, Hull York Medical School, University of Hull, Hull HU6 7RX, UK; 6 Faculty of Medicine, Dalhousie University, Halifax, NS B3H 4R2, Canada

**Keywords:** physical activity, wearable activity monitors, technology, motivation, behavior change, cancer survivors, scoping review

## Abstract

The aim of this review was to explore the acceptability, opportunities, and challenges associated with wearable activity-monitoring technology to increase physical activity (PA) behavior in cancer survivors. A search of Medline, Embase, CINAHL, and SportDiscus was conducted from 1 January 2011 through 3 October 2022. The search was limited to English language, and peer-reviewed original research. Studies were included if they reported the use of an activity monitor in adults (+18 years) with a history of cancer with the intent to motivate PA behavior. Our search identified 1832 published articles, of which 28 met inclusion/exclusion criteria. Eighteen of these studies included post-treatment cancer survivors, eight were on active cancer treatment, and two were long-term cancer survivor studies. ActiGraph accelerometers were the primary technology used to monitor PA behaviors, with Fitbit as the most commonly utilized self-monitoring wearable technology. Overall, wearable activity monitors were found to be an acceptable and useful tool in improving self-awareness, motivating behavioral change, and increasing PA levels. Self-monitoring wearable activity devices have a positive impact on short-term PA behaviors in cancer survivors, but the increase in PA gradually attenuated through the maintenance phase. Further study is needed to evaluate and increase the sustainability of the use of wearable technologies to support PA in cancer survivors.

## 1. Introduction

While cancer remains one of the leading causes of disease burden worldwide, with advancements in early detection and treatment we are seeing more people living longer following a cancer diagnosis [1,2]. As the number of cancer survivors (defined as individuals with cancer from diagnosis to the end of life) continues to grow, additional health concerns are becoming increasingly evident. These include the acute/late effects from the cancer and associated treatment(s), cancer recurrence, second cancers, co-morbid disease, and a multitude of psychosocial issues [1,3,4]. Accordingly, the current challenge for cancer survivorship is to identify novel approaches to help improve overall health and quality of life for survivors.

A growing body of evidence shows that participating in regular physical activity (PA) can lead to physical and emotional improvements for cancer survivors, including but not limited to, improvements in aerobic endurance, muscular strength, self-esteem, functional ability, fatigue, depression, anxiety, and overall quality of life [5,6,7,8,9]. Evidence also indicates that PA confers a survival benefit [10,11]. Despite the positive impact of PA on quality of life and its impact on survival, the majority of cancer survivors do not attain the recommended amount of daily PA (i.e., 150 min of moderate-to-vigorous PA/week or 90 min of vigorous PA/week) [5,12] required to reap these health benefits [13,14,15,16,17].

Learning about preferences and motivations for engaging in PA among cancer survivors is important when developing interventions to change behavior and increase PA. In the past, behavior change interventions have successfully used face-to-face and telephone counselling, email, and print-based methods to increase PA levels among cancer survivors [14,18,19,20,21,22]. However, these methods are often resource intensive, time consuming, and require participants to live near counselling centers; therefore, methods capable of broad reach with low cost are required. With the rapid growth of the internet, access to information has substantially improved and web-based interventions have emerged as the most predominant technology to promote PA behavior change. While several meta-analyses and reviews have summarized the potential utility of web-based technologies for delivering PA interventions amongst both the general population and various chronic disease populations [23,24,25,26,27,28,29], these approaches are not without limitations. A recurring theme in eHealth and mHealth research is poor user engagement and retention [26]. In a recent review of eHealth literature, while it was found that participants engaged with the intervention platform, this engagement decreased over time [30]. Though there are many possible explanations for the difficulties with engagement, one possible explanation may be the inconvenience of using self-monitoring to track activity levels that must be manually entered onto a website. This barrier could be addressed through the use of wearable activity-monitoring technologies. A recent Australian survey indicated that one of the most important characteristics of wearable activity monitors is the ability to automatically sync data, thereby reducing the self-monitoring burden associated with web-based interventions [31].

With increased accessibility, user convenience, continuous monitoring and behavioral feedback, technologies such as wearable activity monitors (i.e., pedometer and accelerometer-based activity trackers) are a promising area in facilitating the delivery of behavioral change interventions designed to promote PA [32]. Wearable technologies (e.g., Apple watch [33], Fitbit [34], Garmin [35]) present data beyond step counts offered by basic pedometers, combined with automated and visual feedback that is lacking with traditional accelerometers. Moreover, these technologies offer several key elements that have been identified as being instrumental for supporting PA behavioral change (e.g., self-monitoring, goal-setting, prompting, social support, social comparison, and rewards) [36,37]. Although wearable PA monitoring technologies are commonly used in research to objectively track PA behavior, fewer studies have explored their potential to motivate and help sustain behavioral change. The overall aim of this scoping review was to explore the acceptability, opportunities, and challenges associated with wearable activity monitors to increase PA behavior in cancer survivors. While there are previous reviews, see Singh et al. 2022, the novelty of our study is that we include two additional years of more recent studies and an analysis of acceptability, opportunities and challenges [38].

## 2. Materials and Methods

### 2.1. Study Design

The use of wearable activity-monitoring technologies in promoting active lifestyles (versus simply monitoring PA) is a rapidly growing field of study, thus a scoping review of the available evidence was conducted to synthesize and map the current state of knowledge and to identify innovative practices, implementation challenges, and gaps in the literature to inform future research and practice [39]. The five-stage framework as outlined by Arksey and O’Malley [39] and Levac et al. [40] was applied. The review stages included: (1) identification of the research questions; (2) identification of relevant articles; (3) selection of relevant articles for review; (4) charting the data; and (5) collating, summarizing and reporting the findings.

### 2.2. Identification of the Research Question

This scoping review aimed to address the following questions:

1. What is the scope and acceptability of the use of wearable activity monitors for individuals with cancer?
2. Does the use of a wearable activity monitor motivate gains in PA behaviors in cancer survivors?

### 2.3. Identification of Relevant Articles

A search strategy was developed and implemented to identify literature relevant to the use of wearable activity-monitoring technologies targeting cancer survivors for promoting active lifestyles. Using an iterative process, keywords were identified and combined around the three components of the research objective: (1) population; (2) wearable technology; and (3) behavior. A description of the keywords used can be found in Table 1. Keywords were searched using Boolean operators to maximize search results. The following databases were used to search the literature: Medline, Embase, CINAHL, and SportDiscus. Databases were searched for English language articles using the identified keywords between 1 January 2011 and 3 October 2022. The search was limited to original research published in peer-reviewed scientific journals.

### 2.4. Selection of Relevant Articles for Review

Article titles and abstracts were independently screened by three authors (MSM, CF, XY) for inclusion in the review. Any discrepancy was settled by consensus. Research articles were required to focus on adults (18+ years) with a history of cancer and include the use of a wearable device that objectively monitored PA behavior with the intent to motivate behavior change through the provision of relevant feedback to the user (e.g., number of steps taken, sitting time, movement prompts). Research protocols and studies that focused on monitoring behavior but did not include individual feedback, including features to motivate behavior change, were excluded from the review.

### 2.5. Charting the Data

Using an iterative process, data from the search results were extracted onto a data abstraction form. A descriptive analytical approach [39] was used to extract, synthesize, and share the data for team review. Extracted data included: (1) authors and year of publication; (2) objectives; (3) study design/overview of methods; (4) type and duration of intervention; (5) key outcome measures; and (6) key findings. Study quality assessment was conducted using the Cochrane risk of bias assessment [41]. The elements used for the assessment included: (1) sequence generation, (2) allocation concealment, (3) blinding of participants and personnel for all outcomes, (4) blinding of outcome assessors for all outcomes, (5) incomplete outcome data for all outcomes, (6) selective outcome reporting, and (7) other sources of bias [38,42,43]. With the maximum risk score of 7, a score of 0–2 indicated low risk, 3–5 indicated moderate risk, and 6–7 indicated high risk. The quality assessment was conducted by a co-author (XY).

The key outcomes of interest were changes in PA, retention rate, and perceived acceptability. Findings were also summarized in the context of study quality to gauge the risk of bias and understand the strength of the evidence provided. Some of the challenges in appraising this broad evidence base include the predominance of pre-post studies, and lack of blinding in RCT studies.

## 3. Results

**Figure 1:**
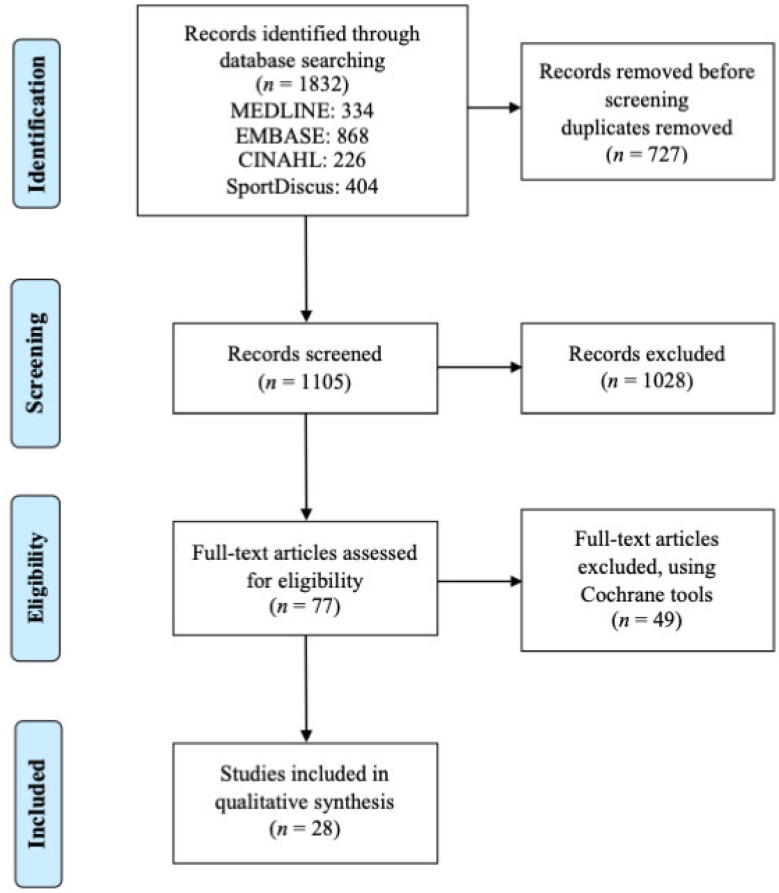
The Preferred Reporting Items for Systematic reviews and Meta-Analyses flow chart.

The search resulted in a total of 1832 published articles with 1804 excluded based on review of the articles and application of inclusion/exclusion criteria at progressively more detailed levels (i.e., review of article title, abstract, full manuscript), see Figure 1.

A total of 28 original research articles met the inclusion criteria and were deemed eligible for the scoping review. A summary of original research is presented in Table 2.

**Figure 2:**
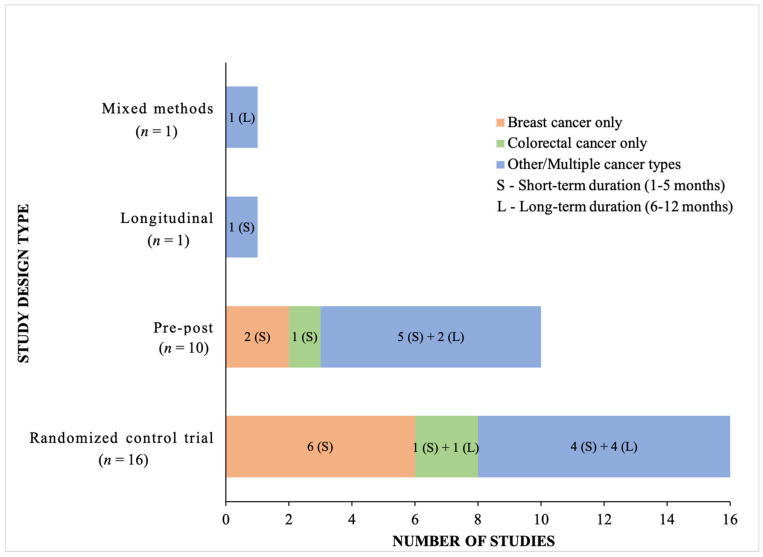
Study designs tabulated by cancer type and duration of intervention.

Of the 28 original articles included in the review, over half were randomized control trials and one third were pre-post designs (see Figure 2). The most common cancer type was breast cancer (eight studies) followed by colorectal cancer (three studies), with the remaining studies including a handful of other individual cancers (peritoneal cancer, endometrial cancer, ovarian cancer, lung cancer) or multiple cancer types. The duration of interventions included short term interventions (defined as those lasting one to five months) and longer-term interventions (defined as those lasting six to 12 months), Figure 2. Eighteen studies included post-treatment cancer survivors, eight included those on active cancer treatment (five were pre-surgery), two included all phases of cancer treatment.

To measure PA as an outcome, accelerometers were the primary device used in 46% of the original studies. Of the remaining studies, 4% used pedometers and 41% used a combination of self-monitoring devices to assess changes in PA, including Fitbit [45,46,52,53,54,55,56,57,59,63,65,66,68,71], Neofit [58], Garmin Vivofit [60,61], Xiaomi wristband [70], treadmill and heart rate monitors [44,47,49,50,58,69].

Of the original studies reviewed, 25 reported some type of PA outcome (i.e., number of steps, overall PA, walking time, activity intensity, etc.). All but three studies reported that the use of wearable activity-monitoring devices increased participant levels of PA. It should be noted that although the three studies did not find an increase in participant PA levels, they did observe a slower rate of PA decline in the participants using the pedometers. Similarly, the studies that did not find an increase in PA in participants using activity monitors, reported that the monitors may have played a role in maintaining study adherence [52,57].

Adherence (or retention) rate was used to assess the acceptability in almost all studies (96%). With one exception, these studies all reported high adherence rates and therefore indicate good acceptability of the technology. Questionnaires were used to measure satisfaction in 50% of the studies, with 14% requesting further feedback and 36% performing additional semi-structured interviews.

Using the Cochrane risk score, see Table 2, 15 of the studies (54%) were rated as low quality, 10 (36%) were rated as moderate quality, and three (11%) were rated as high quality. Sequence generation was the quality component most frequently rated as high risk. In most pre-post studies this was a consequence of the lack of comparison groups. In general, most studies lacked blinding due to the nature of the intervention (i.e., a wearable monitoring device).

## 4. Discussion

As the majority of cancer survivors are not sufficiently active to attain the associated health benefits, motivating survivors to adopt and maintain a physically active lifestyle remains a considerable challenge [72]. Accordingly, novel approaches to foster and maintain PA are urgently needed. This review highlights that wearable devices are important tools to help promote and increase PA in cancer survivors, while also emerging as valuable tools to raise awareness in individuals of their own activity levels. Hence, wearable devices present valuable opportunities for improving the health of cancer survivors, through overall increases in PA. While interventions to change PA behavior have employed several strategies with variable success, active self-management approaches (i.e., engaging the individual in their own behavioral change) were found to be effective [73]. Specifically, the most successful interventions are those that have employed techniques to elicit behavioral change. Although the self-monitoring device-derived PA increases were typically robust and steady at initiation, generally activity rates decreased throughout the maintenance phase. For instance, several studies reported independent effects of self-monitoring wearable devices increasing regular exercise [64,66,68], reducing sitting time [60] and increasing adherence to PA guidelines [48,55] in the first three months. Despite this, there were very few studies examining the maintenance of these effects long-term. Lynch et al. [61] reported an abbreviated increase in PA during the following 3-month maintenance period. Similarly, studies found the adherence rate for wearing self-monitoring devices declined after three months [46] and 12 months [70]. Future studies are required to assess the maintenance of device-driven increases in PA.

While as many as 93 distinct behavior change techniques have been recognized, several systematic reviews have identified a smaller number of techniques that are associated with effective PA interventions [74]. For instance, Michie et al. [73] found that of the self-regulatory techniques reviewed, self-monitoring was the most important. They also found that combining self-monitoring with at least one additional self-regulatory technique (e.g., goal setting, feedback on performance) was associated with improved intervention effectiveness. Similarly, a meta-analysis by Bravata et al. [75] found that while the use of pedometers improved walking behaviors, the benefits were limited to those studies that used pedometers in conjunction with additional supportive behavioral change techniques. For example, the addition of a daily activity record has been found to improve intervention effectiveness as it provides a record of success, additional feedback on an individual’s behavior patterns, identifies areas for improvement, and assists with personalized goal setting [51,55]. Marthick et al. [62] also reported a higher retention rate as a result of additional individual input on the PA. The results of this current scoping review are consistent with these findings, noting that while activity monitors proved effective to foster positive change in walking behaviors, they were not used in isolation. Few of the reviewed studies [46,50,60,61,66] explicitly emphasized the use of behavior change techniques; however, when used in conjunction with these techniques (i.e., self-regulation, self-efficacy, modelling, social support, etc.), the studies reviewed herein provide additional evidence that pedometers, and other activity monitors, offer an accessible, user friendly (i.e., low-tech/low-literacy), real-time performance feedback tool that fosters PA behavior change.

The perceived acceptability was manifested by positive results in satisfaction [46,65] and compliance [57,58] rates. The majority of participants were willing to spend time installing the relevant software [49] and to continue using the wearable devices after the intervention ended [58]. Accordingly, several studies reported that feedback was particularly beneficial for increasing PA, with the majority of research participants reporting that text messages and self-monitoring data had a motivating effect [51,68,71]. Further, Groarke et al. [53] reported a very positive user experience during post-surgery, which was associated with general psychological well-being. Likewise, qualitative interviews from Marthick et al. [62] noted that most participants found the activity monitors (Misfit Shine) stimulating regardless of accuracy problems, and they enjoyed the sense of achievement that comes from receiving personalized messages. Although the qualitative analysis had limited generalizability and significant potential for selective result reporting, they revealed analogous trends to the quantitative findings. It is clear that the acceptability of the wearable devices was high, which highlights the valuable opportunity these devices present for supporting interventions aimed at increasing PA.

Activity monitors have been shown to be a valuable tool in improving awareness of activity levels and providing additional motivation to improve PA behaviors; however, they are not without limitations. For example, while providing a reasonably accurate estimation of PA level through a measure of step count, activity monitors such as pedometers are not able to detect non-ambulatory activities such as cycling, weight training, or swimming. Likewise, basic activity monitors are not able to give a measure of overall activity intensity [76]. Importantly, while generally increasing the amount of daily movement (i.e., steps per day) is an effective way to reduce the effects of sedentary behavior, activity intensity must be at least moderate to achieve optimal health and fitness benefits and should thus be monitored [77]. Although activity monitors, such as accelerometers, are able to measure activity intensity, their use is often limited to research settings where the data can be downloaded and appropriately interpreted [76]. It is also worth noting that blinding was nearly impossible during the interventions owing to the obvious presence of technology. Hence, there was a strong possibility that individuals in the intervention groups maintained higher levels of PA because they were aware that they were being observed. However, being observed, or gaining feedback, was found to increase PA, which is a positive outcome.

Newer generation wearable activity monitors have improved upon traditional pedometers and accelerometers through the addition of an interactive, user-friendly, mobile interface that provides a visual representation of real-time data [78]. Importantly, recorded data can be wirelessly synced to a mobile device (e.g., smartphone) or computer for long-term data storage, detailed behavior tracking, and personalized feedback; foregoing the need for manual tracking and data input required by traditional pedometers. Moreover, many of the newer activity monitors capture data beyond PA (e.g., heart rate, sleep, sedentary time) and can be synced with companion web-based or mobile apps that offer additional tools to track and offer supplementary feedback on related lifestyle behaviors (e.g., diet, sleep, stress), provide health education, and model/demonstrate target behaviors [37,79,80]. Likewise, sophisticated algorithms continue to advance the overall utility of the captured data by further personalizing activity and related health goals. For example, “Personal Activity Intelligence” uses heart rate data collected by an activity monitor and personal information (age, sex, heart rate reserve) to develop a personalized activity score that is used to guide the individual on the amount of exercise needed to decrease the risk of death from cardiovascular disease [81]. Importantly, activity monitors and their associated apps include several evidence-based behavioral change techniques known to be associated with successful behavioral change (e.g., self-monitoring, goal-setting, behavioral feedback and prompts, social support, social comparison, rewards, provision of health information and instruction) [37,79]. As outlined in this and similar reviews, there is a growing body of literature that demonstrates the feasibility and potential utility of both traditional and newer generation activity monitors in fostering PA behavior change in individuals with a chronic illness [37,79,82,83,84,85].

Despite their documented promise to help facilitate PA behavior change, the implementation of wearable technologies is not without challenges. While a major strength of such devices is the wealth of individual level data that is captured (e.g., behavioral and physiological), issues of data management have emerged. For example, the personal and health data associated with wearable activity monitors are often collected and stored by the manufacturer raising concerns about privacy, the security of the data itself, and data ownership; with the possibility of data being shared with third parties [85]. As many technologies involve automatic uploading of personal data to a central server for processing to provide the necessary feedback to users, ensuring that personal data remains private and not available for sale to third party companies without explicit consent is crucial. Thus, ethical, legal and social issues related to data ownership and data access need to be further explored.

These technologies ultimately need to be widely adopted to realize the full potential of wearable activity monitors. However, the fear of use of new technology may inhibit some individuals from embracing wearable activity monitors, and challenges related to internet access, mobile device use or cost may be issues for some people. Consequently, a move towards the use of high-tech wearable devices in cancer control activities may disenfranchise those individuals who are wary of, or dislike technological devices, or face other barriers to access. Interestingly, while older adults are often slower in adopting new technologies, studies have shown that younger adults appear to use activity monitors to improve fitness, whereas older adults have adopted their use to help improve overall health [85]. Likewise, while individual preferences for the use of technology may vary, data shows that user expectations and overall usability (e.g., user-friendly, visual display, automated feedback, overall comfort, data syncing, data accuracy, etc.) are also key to promote overall acceptability and feasibility of use [85,86]. Furthermore, the impacts of several challenges in cancer survivorship care, such as financial cost, internet access, and reading skills, have yet to be explored in the studies we reviewed, whereas these underlying inequalities are paramount in public health.

## 5. Conclusions and Future Directions

The increasing availability and relative low cost of activity monitors is leading to a rapidly growing consumer base [87]. Studies included in this review yielded generally positive results in self-efficacy, coherence, and perceived utility, and both quantitative and qualitative findings show that self-monitoring wearable technologies increase PA. While these effects were typically robust and steady at initial use, they gradually reduced throughout the maintenance phase. Further study is needed to evaluate the sustainability of wearable activity monitor interventions to support PA increases.

## Figures and Tables

**Table 1:** Scoping review search strategy.

Search	Review Component	Query Terms
1	Population	cancer OR tumor OR malignancy OR neoplasm
2	Wearable technology	monitor OR wearable technology OR wearable device OR Fitbit OR Garmin OR Apple OR Polar OR Huawei OR Samsung
3	Behavior	physical activity OR exercise OR fitness OR physical exercise OR sedentary OR sitting
4	All	#1 and #2 and #3

**Table 2:** Summary of articles included in the review.

Author	Objective	Study Design/Methods	Activity Monitor/Intervention	Key Outcome Measures ^†^	Key Findings ^†^	Risk of Bias Score
Brown et al. (2018) [44]	Characterize changes in circulating tumor cells after exercise training	RCT. Participants (*n* = 23) were randomized to either low-dose (*n* = 11), high-dose (*n* = 7) or usual care (*n* = 5). Baseline and 6-month assessments.	Control: usual care; Intervention: low-dose (141 min/wk), or high-dose exercise (247 min/wk). Activity monitor: in-home treadmill, and heart rate monitor.	Adherence rate was used to measure the feasibility.	Over six months, the low-dose group had an adherence of 93%, and the high-dose group had an adherence rate of 95%.	0/7
Cadmus-Bertram et al. (2019) [45]	Test the feasibility of augmenting care planning with a multi-level PA intervention	RCT. Participants (*n* = 50) were randomized to intervention (*n* = 26) or comparison (*n* = 24). Baseline and 12-weeks assessments.	Control: dietary guidelines + standardized email contact; Intervention: care plan + Fitbit-based PA module + in-persona session with goal-setting + email-based coaching + Fitbit review. Activity monitor: ActiGraph GT3X+ accelerometer.	PA was accelerometer-measured by MVPA, and daily step. Clinicians and support partners reviewed web-based surveys within EHR that track frequency and usefulness of exercise process.	Participants and their support partners experienced substantial increases in accelerometer-measured PA after completing the intervention session. In terms of EHR online feedback, half (50.0%) rated the procedure as “very easy”, 18.2% as “very easy”, 13.6% as “neither easy nor difficult”, and 18.2% as “somewhat difficult”.	2/7
Chan et al. (2020) [46]	Determine the feasibility and acceptability of a remotely delivered web-based behavioral intervention among men with prostate cancer.	RCT. Participants (*n* = 202) were randomized to either level 1 (*n* = 49), level 2 (*n* = 51), level 3 (*n* = 50), or level 4 (*n* = 52). Baseline, 3- and 6-mon assessments.	Control: L1 = general educational information, resource directory, and study-specific guidelines. Intervention: L2 = L1 + personalized diet and exercise prescriptions. L3 = L2 + Fitbit Alta. L4 = L3 + 2 optional calls from either exercise trainer or registered dietician. Activity monitor: Physical activity reported by Fitbit	The effect of intervention was evaluated by self-reported diet and PA. The accrual time and retention were used to determine feasibility. Personal surveys were used to assess acceptability with general satisfaction.	The follow-up rate at 3 months was 82.7%, whereas it was 77.2% after 6 months. The intervention was well received by the vast majority of responders. The highest percentage of Level 4 participants were highly satisfied. Level 1 expressed the most dissatisfaction. At 3 months, there was a minor difference in diet and PA between males in level 4 and those in level 1.	2/7
Cheong et al. (2018) [47]	Evaluate the efficacy and feasibility of comprehensive mobile health care of colorectal cancer patients during active chemotherapy	Pre-post design. Assessments were conducted in baseline (*n* = 102), 6-week (*n* = 92), and 12-week (*n* = 75).	Intervention: smartphone aftercare program + wearable device (Internet of Things) + rehabilitation exercise. Activity monitor: Self-reported questionnaire and wearable device.	Subjective measurement: Questionnaires track the amount of time spent exercising MVPA and walking. Objective measurement (wearable device): number of steps, walking distance, and heart rate. The feasibility was determined by the compliance rate.	Although the quantity of PA dropped from 6 to 12 weeks, the overall amount of weekly PA improved after 12 weeks. The participants’ compliance rate was 83.8%, while the rate of dizziness and dyspnea during exercise was 15%.	6/7
Ferrante et al. (2022) [48]	Examined adherence with a physical activity tracker and patterns of activity among different subgroups of African American/Black breast cancer survivors	This is a follow-up (*n* = 44) to an RCT consists of control (*n* = 17), intervention (*n* = 17), an additional intervention (*n* = 10). Baseline, 3-, 6-, 9-, and 12-month follow-up visits.	Control: Fitbit only; Intervention: SparkPeople + Fitbit; Additional intervention: SparkPeople Premium + Fitbit; Physical monitor: Fitbit Alta. Self-reported questionaries on self-regulation and self-efficacy	Fitbit devices calculate active minutes for activities lasting at least 10 min at or above 3 METs. Insufficient Fitbit wear or data capture (fewer than 1000 steps) were deemed non-adherent.	Adherence was shown to be significantly related to steps and active minutes. Activity levels were significantly correlated to self-monitoring, goal setting, and self-efficacy. Some subgroups, such as those over 60, retired, with a BMI more than 40, a greater number of comorbidities, or more household members, may demand further assistance.	2/7
Finley et al. (2020) [49]	Explore the feasibility, acceptability and perceived utility of a wearable fitness device and an exercise prescription from a surgeon	Pre-post design. Assessments were conducted in baseline (*n* = 30), and post-study (*n* = 17). Day of surgery, 2- and 16- weeks after surgery, and two semi-structured interviews.	Intervention: prescription for 150 min/wk MVPA exercise. Activity monitor: Garmin Vivoactive HR device. Alternatively, they can use their own.	Acceptance is defined as (a) the number of days that any form of information from the device was received and (b) the number of days that heart rate data was specifically received. Semi-structured phone interview on the acceptability of the device and exercise prescription. Participants may self-motivate by viewing their heart rate time series every 15 s, number of steps taken, floors climbed, minutes spent exercising, and near real-time GPS locations.	Acceptance: During the pre-operative phase, 71% of registered participants successfully synced their device. During the post-operative period, 75% were active. Perceived utility: Ten individuals (36%) expressed satisfaction with the device. They enjoyed how the device provided them feedback on their activity level (*n* = 6) or progress over time (*n* = 2), and how it reminded them to move through alert system (*n* = 2).	5/7
Gehring et al. (2018) [50]	Investigates the feasibility of a home-based, remotely guided exercise intervention for patients with gliomas.	Randomized controlled trial. 34 (*n* = 202) were randomized to either control (*n* = 11) or intervention (*n* = 23). Baseline and 6 months assessments.	Intervention package: individualized exercise prescription + weekly personal feedback by e-mail + last call on the feedback on program. Control: motivational brochures + bi-monthly phone calls on general health questions. Activity monitor: Objective measurement by heart rate monitors. Self-reported PA by the *International Physical Activity Questionnaire*	Adherence was measured as the proportion of physical exercise sessions performed throughout the time out of the specified sessions. The average heart rate of all training sessions as a percentage of the maximal heart rate as determined during the first exercise test showed average intensity. Two physiotherapists independently rated overall exercise performance for each participant.	The average adherence to scheduled sessions was 79%. Patients had positive experiences. There were no negative events. The physical exercise program was rated as satisfactory or good by 84% of participants. The exercise group improved more than the control group in maximal cardiopulmonary exercise testing (+158.9 mL/min; 95% CI: 44.8 to 362.5) and BMI (0.3 kg/m2; 95% CI: 0.9 to 0.2).	0/7
Gell et al. (2017) [51]	Examine the efficacy, feasibility, and acceptability of a technology-based intervention to promote maintenance of PA	Pre-post design. Baseline (*n* = 38); post study (*n* = 24). Participants meet weekly to download/review weekly PA by step data for Fitbit step counts and minutes.	Intervention package: tailored text message + Fitbit self-monitoring, and brief health coaching sessions. Physical Monitor: Fitbit and Actigraph GT3X+ accelerometer. Fitbit was used to support self-monitoring and text message content. Locations were assessed by QStarz BT-Q1000XT GPS.	MVPA were estimated by total weekly minutes and average daily step counts. Acceptability was determined by the percentage cancer survivors who agreed to participate in the study, intervention attrition and post-intervention questions on satisfaction.	The intervention was well-received by the majority of participants, with 87% satisfied with the health coach and Fitbit and 91% satisfied with the text message content. The majority of participants reported that text messages and Fitbit improved their motivation and the quantity of PA they performed. There was no attrition among those who began the intervention. There was no significant difference in PA levels measured with an accelerometer before and after the intervention, suggesting that PA levels were maintained 4 weeks after completing exercise-based cancer rehabilitation.	3/7
Granger et al. (2018) [52]	Determine the: (1) feasibility and (2) exploratory effectiveness of a PA self-management program aiming to increase PA levels	Pre-post design. Baseline (*n* = 42) and 8-weeks follow-up (*n* = 37) assessments. Followed-up with weekly telephone consultations.	Intervention package: unsupervised home aerobic exercise program + written information manual + Fitbit for self-monitor and personalized program/goals. Activity monitor: The Fitbit was used to self-monitoring behavior, but PA was self-reported by questionnaire.	The feasibility was determined by the rate of consent and the number of consultations provided. As a result, a 70% consent rate determined viability. The secondary assessment focused on the start date of the intervention, the number of intervention consultations provided, and the location of the initial consultations.	Participants undergoing lung cancer surgery may be interested in such an intervention (89% consent rate), are able to participate when it is delivered in the post-surgical setting (100%) and may not have declines in PA levels as a result. There were no statistically significant changes in self-efficacy for PA.	6/7
Groarke et al. (2021) [53]	Examine the acceptability of a behavior change intervention using mHealth for cancer survivors with a BMI of 25 or more.	RCT-based mixed method: 13 participants were interviewed, and 36 participants completed the quantitative survey. At the 24-week follow-up.	Intervention: 8-week PA goal setting: Fitbit activity monitor + SMS contact. Activity monitor: Fitbit.	Qualitative: Semi-structured interviews were used to assess retrospective acceptance. Quantitative: Response rates and retention rates were also used as measures of the acceptability of the intervention.	The majority of survey respondents (35/36, 97%) were satisfied with the intervention. Many of the intervention components were liked in qualitative reports, with the mHealth components receiving particularly positive ratings. The burden of participation in the intervention was rated as either high (6/36, 17%) or low (5/36, 14%). The majority of respondents (35/36, 97%) reported they understood how the intervention worked, and qualitative data show that participants’ understanding of the intervention’s goal was broader than weight control and focused more on moving on psychologically after cancer.	5/7
Hardcastle et al. (2020) [54]	explore patterns of Fitbit-measured PA and wear-time over 24-weeks and their relationship to changes in Actigraph-derived moderate-to-vigorous PA (MVPA).	This is a follow-up to an RCT. Pre-post design. Baseline (*n* = 29) and post-study (*n* = 28). Baseline, intervention (12-weeks) and end of follow-up (24-weeks) assessment.	Intervention: Fitbit Alta + two-hour group sessions + action-planning and goal-setting + phone-call feedback. Daily steps and active minutes were recorded. Activity monitor: The Actigraph GT9X research grade accelerometer provided minutes/week of MVPA. The Fitbit Alta was used for self-monitoring and explore patterns of PA.	Fitbit wear-time adherence rates (percentage of valid wear days). As a result, a valid wear-day was defined as a step count of =>1000 steps each day. Participants performed daily accelerometer diaries to allow for data cross-checking. For uniaxial and triaxial cut point definitions, MVPA was defined as =>1952 and =>2690 counts per minute, respectively.	The median adherence score for all 24 weeks examined separately was 100%. Fitbit wear-time was also high during the follow-up period (13 to 24 weeks), with an adherence score of 98%. (IQR 75 to 100).	2/7
Hartman et al. (2018) [55]	Examine patterns of Fitbit use and activity and their relationships with success in the intervention based on ActiGraph-measured MVPA.	Pre-post study to follow an RCT. Baseline (*n* = 43) and post-study (*n* = 42) assessment.	Intervention package: Fitbit for self-monitoring PA + phone calls (2-week and 6-week time points and automatic emails every 3 days throughout the 12-week intervention, which included reminders to sync and wear their Fitbit). Activity monitor: Fitbit. ActiGraph GT3X+ accelerometer.	Active Minutes and daily adherence were measured by Fibit tracker. Accelerometers were used to determine frequency, duration, and intensity. Self-report questionnaires were utilized to measure how frequently individuals glanced at their Fitbit tracker activity data.	Adherence to wearing the Fitbit was robust and consistent, with a mean of 88.13% of valid days (SD 14.49%) for 12 weeks. Greater adherence to Fitbit use was related to higher increases in MVPA. The highest minutes of MVPA occurred at week 3, immediately after the intervention call, which generally occurred towards the end of week 2, and at week 9, which was approximately when participants were contacted to confirm their final visit at 12 weeks.	2/7
Kanzawa-Lee et al. (2022) [56]	Explore the effect of an 8-week home-based brisk walking (the “MI-Walk”) intervention compared with PA education alone.	RCT. Participants (*n* = 57) were randomized to control (*n* = 28) or intervention (*n* = 29). Baseline and 8-month assessments.	Control: PA education and phone assessments. Intervention: control + MI-WALK motivational supports (Fitbit + enhancement therapy session). Activity monitor: Fitbit.	A 0–793 scaled questionnaire was used to assess self-reported PA. Questionaries were used to assess self-reported motivational interviewing fidelity and PA.	The intervention and control groups had the same self-reported PA scores at baseline (*n* = 51) and 8 weeks (*n* = 48). Among the MI-Walk intervention participants, no baseline Fitbit data (only post-intervention initiation) were gathered. As a result, there was no analyses on the change in PA.	2/7
Kenfield et al. (2019) [57]	Determine the feasibility and acceptability of a digital lifestyle intervention among men with prostate cancer.	Randomized controlled trial. Participants (*n* = 76) were assigned to either intervention (*n* = 37) or control (*n* = 39). Baseline, first 12-week, and second 12-week assessments.	Control: only usual standard of care; Intervention: personalized recommendations on website + Fitbit One + and text messaging. Activity monitor: Self-reported questionaries on PA and accelerometers.	The rates of recruitment and the utilization of study components were used to determine feasibility. Adherence was determined each week as responding to a text message. Online questionnaires with closed and open-ended questions were used to measure acceptability. After 12 weeks, Fitbit activity data was used to examine responses on the text messaging platform, and website login and page visit data were used to quantify study component consumption.	The intervention arm self-reported change was 1.1 h per week (IQR: −0.3, 3.6), while the control arm was 0 h per week (IQR: −1.1, 1.7). There were no statistically significant differences between the groups. Accelerometer: There were no differences in step counts, moderate, or MPVA between the two arms. Participants in the intervention wore their Fitbits for an average of 82 days (IQR: 72–83), with 98% of the days falling within the 12-week period, responded to an average of 71% of text messages (IQR: 57–89%), and saw the website for an average of 3 days (IQR: 2–5). Acceptability: 60.7% of participants rated the website as high or very high in quality, 87.1% rated Fitbits as good to excellent, 68.8% rated SMS messaging as good to exceptional, and 78.1% rated the baseline personalized suggestion report as good to excellent. Participant satisfaction (defined as “pleased” or “very satisfied”) for the website was 60%, 90.6% for Fitbits, and 73.3% for text messaging.	2/7
Kim et al. (2020) [58]	Evaluate the efficacy and safety of rehabilitation exercises among hepatocellular carcinoma patients.	Pre-post design. Baseline (*n* = 37); post study (*n* = 31). Baseline, and 6- and 12- week assessments.	Intervention: Neofit (wearable wristband) + mHealth app + prescribed rehabilitation exercise. Activity monitor: Neofit.	Neofit assessed the number of steps taken, calorie expenditure, activity time, and heart rate using wearable sensors. Self-reported MVPA minutes per day or days per week, as well as time spent walking or sitting in the previous 7 days. Physical fitness was carefully assessed using clinical equipment.	The completion rate for this trial was 84% (31/37). According to a satisfaction survey, after 12 weeks of mHealth program for patients, 84% of participants rated medium-to-high satisfaction with the program. After the trial, 87% of participants stated a wish to continue utilizing the program. Both the 30-s chair stand test and the 6 min walk test significantly improved from 0 to 6 weeks, 0 to 12 weeks, and 6 to 12 weeks. Muscle mass and the IPAQ-SF score increased considerably after 12 weeks of therapy, with no biochemical deterioration.	4/7
Low et al. (2020) [59]	To develop and test a mobile technology-supported intervention to reduce Sedentary behavior before and after cancer surgery, and to evaluate the usability and feasibility of the intervention.	Pre-post design. Baseline (*n* = 15); post study (*n* = 14). Participants were called once per week to complete semi-structured interviews during the 30 days after hospital discharge	Intervention: Fitbit + a smartphone app (i.e., Detecting Activity to Support Healing) + weekly call + semi-structured interviews. Activity Monitor. Participants were asked to respond to the activity prompt. The prompt was calculated by their Fitbit PA data.	(1) Weekly ratings on how easy it was to use each app’s interface in terms of appearance design, and usability; and how satisfied the participant was overall with the DASH intervention program. (2) A ten-item questionnaire based on the Usability Scale. The semi-structured interviews’ notes were examined and organized into recognized themes. Accrual and retention rates, as well as compliance with reporting symptoms, were used to determine feasibility. A questionnaire about the usability of the apps. Semi-structured interviews were used to assess the intervention.	Low (1/15, 7%) attrition was due to poor health and extended hospitalization. Fitbit compliance was 70% (653/927 days) overall, however it decreased from before surgery (330/364, 91%) to inpatient (51/143, 36%) and post discharge (272/420, 65%). Fitbit wear time compliance is also dropping, which is consistent with research in healthy individuals, which found that 40% of participants abandoned the Fitbit after six months. Overall system satisfaction was 89.9, while the mean System Usability Scale score was 83.8 out of 100.	4/7
Lynch et al. (2019) [60]	Determine the efficacy of a 12-week intervention for increasing MVPA and reducing sedentary behavior for postmenopausal breast cancer survivors.	RCT. Participants (*n* = 83) were assigned to either intervention (*n* = 43) or control (*n* = 40). Baseline, first 12-week, and second 12-week assessments.	Control: Behavioral feedback and goal-setting session + telephone-delivered behavioral counseling. Intervention: control + Garmin Vivofit 2 (activity monitor). Activity monitor: Actigraph GT3X+ accelerometer, Garmin Vivofit 2, and activPAL.	The retention rate was used to measure acceptance. Accelerometer data is used to count movement. MVPA was computed by adding together the average weekly time spent and time spent in “bouts” of 10 min or more. The activPAL counts the number of posture changes as well as the length of time spent in each posture.	The experiment had a high retention rate, with 80 (96%) of patients completing T2 data collection. At T2, there was a statistically significant difference in MVPA across groups (69 min/wk; 95% CI = 22–116; *p* 0.01), favoring the intervention arm. Overall sitting time (37 min/d; 95% CI: 72 to 2; *p* = 0.01) and protracted episodes of at least 20 min length (42 min/d; 95% CI: 83 to 2; *p* = 0.04) were statistically different in the intervention arm.	2/7
Lynch et al. (2019) [61]	(1) to examine the maintenance of MVPA and sitting time changes in the primary intervention group approximately 12 weeks after intervention (T3) (2) to determine the efficacy of an abridged intervention (Garmin Vivofit 2 only).	This is follow-up analysis (T3) after the intervention (T2) for the Lynch-1. For aim 1 (maintenance): Baseline (*n* = 43); post study (36 for maintenance, 30 for sitting). For aim 2: baseline (*n* = 40), post study (*n* = 37). Assessments were T3 after the Lynch-1 (T2).	Intervention and control are the same from above. Activity Monitor: MVPA was measured by the Actigraph *GT3X+* accelerometer, calibrated by the Garmin Vivofit 2. Sitting time was assessed by the activPAL. Garmin Vivofit 2 was used to assess the MVPA in Aim 2.	The retention rate was considered to assess the acceptability. MVPA was quantified using the Sasaki vector magnitude cut point (using tri-axial data) of 2690 counts per minute (we also used the Freedson and Matthews cut points); sitting time was measured using the activPAL, which participants were instructed to wear 24 h a day.	The retention rate was 87%. The study had a good retention rate, with 80 (96%) of individuals completing T2 data collection. (95% CI = 18 to 46; *p* = 0.37); the mean change between T2 and T3 was 8 min per week (95% CI = 17 to 33; *p* = 0.52). At T3, the MVPA of participants in the primary intervention arm was 86 min per week (95% CI = 47–125; *p* 0.01) greater than at T1. At T3, participants had increased their MVPA by 33 min per week (95% CI = 3–64; *p* = 0.03). The average increase in MVPA from T1 was much larger (38 min/week, 95% CI = 4–73; *p* = 0.03). In the waitlist control arm, average sitting time was decreased by 38 min per day (95% CI = 69 to 7; *p* = 0.02), but sitting time at T3 was only 23 min less than at T1 (95% CI = 54 to 8; *p* = 0.15). Between T2 and T3, the abbreviated intervention reduced prolonged sitting by 28 min per day (95% CI = 60 to 5; *p* = 0.09).	5/7
Marthick et al. (2018) [62]	Evaluate the feasibility, usability, and acceptability of an interactive Web portal developed to support patients with cancer to increase daily PA levels.	longitudinal cohort design. All participants (*n* = 49) were allocated to 3 cohorts: 1. Web portal (*n* = 17) 2. Web portal + summative messaging (*n* = 17) 3. Web portal personalized coaching messaging (*n* = 15).	Intervention: Interactive web portal, included integration of real-time wearable activity device data, collection of PROs and symptom information, the provision of educational material, and individualized coaching messaging to support behavior change by encouraging patient engagement in PA. Activity monitor: Misfit Shine activity monitor or Fitbit was used to measure feasibility.	To measure feasibility, the number of log-ins and completed surveys were employed. Semi-structured qualitative interviews were used to assess acceptability, which included participant satisfaction, acceptability of the intervention, self-efficacy linked to changes in lifestyle determinants, median daily step count, and weekly email involvement.	The number of individuals satisfying the two feasibility criteria grew over the cohorts, with cohort 1 having the fewest (7/17, 35%) and cohort 3 having the highest (12/14, 86%). Only cohort 3 satisfied the feasibility criterion. The activity tracker distributed to participants was generally well received, with individuals indicating that they enjoyed it and found it straightforward to use. Participants were extremely or somewhat happy with the intervention, with 83% (33/40) of responders extremely or moderately satisfied. Satisfaction with the Misfit Shine activity tracker was high, with 77% (31/40) of respondents extremely or fairly satisfied.	5/7
Maxwell-Smith et al. (2019) [63]	Ascertain whether activity monitor coupled with action planning was effective in increasing PA in colorectal and endometrial cancer survivors at cardiovascular risk.	RCT. All participants (*n* = 68) were randomized to intervention (*n* = 34) or control (*n* = 34). 30 min assessments at baseline and 12 weeks.	Control: PA guidelines. Intervention: Fitbit Alta + group session + support phone call. Activity monitor: Fitbit and ActiGraph Link GT9X accelerometer.	The ActiGraph was also used to calculate the number of minutes per day of MVPA accumulated in bouts of at least 10 min. The wristworn Fitbit Alta tracker was utilized as an experiment to capture daily steps. The ActiGraph GT9X accelerometer was used to calculate MVPA minutes each week. MVPA is accumulated in 10 min increments) and distance, and it gives automated alerts pushing participants to collect 250 steps each hour.	With 94% attendance across group sessions, intervention adherence was excellent. The majority of intervention group participants (88%, *n* = 29) accepted the Fitbit friend invitation. Fitbit involvement was high, with 86% (SD = 29) of valid weardays recorded throughout the 12-week period (*n* = 28). A legitimate wearday was defined as a step count of 1000 steps each day. The intervention group raised MVPA by 45 min per week, whereas the control group decreased by 21 min per week. On both triaxial (29 vs. 8 min/wk) and uniaxial (31 vs. 7 min/wk) measurements of MVPA accumulated in bouts of at least 10 min, the observed mean increases in MV10 were higher in the intervention group compared to controls one.	1/7
Rastogi et al. (2020) [64]	Determine whether the PA module improved lasting behavior change.	RCT. Participants (*n* = 50) were randomized to intervention (*n* = 26) or control (*n* = 24). Baseline and 12-weeks.	Control: Dietary guidelines + standardized emails. Intervention: Fitbit tracker + customized email feedback. Activity monitor: ActiGraph GT3X+.	The scale runs from 1 to 5, with higher scores suggesting that the person is working to improve their PA-related thoughts and behavior. Decision- making balancing was used to investigate the perceived benefits and barriers of PA.	A total of 94% of survivors retained in the study after 12 weeks. The intervention was associated with moderate-to-large improvements in physical health (effect size: d = 0.39, 95% CI = 0.0, 0.78), mental health (d = 0.59, 95% CI = 0.19, 0.99), sleep impairment (d = 0.62, 95% CI = 1.02, 0.22), and exercise self-efficacy (d = 0.60, 95% CI = 0.20, 1.0) compared to the controls.	2/7
Schrier et al. (2021) [65]	(1) assess the feasibility and acceptability of the intervention. (2) assess the change in mean daily step counts.	Pre-post design. Baseline (*n* = 29) and post-study (*n* = 24). Baseline 2-week intervention; 12-week follow-up.	Intervention: two Fitbit Charge 2 s (one for the participant and one for the teammate). Fitbits + increased step goal + collaborative game integrates wireless devices, clinical trial randomization and enrollment processes, self-administered surveys, automatic transfers of financial incentives, and secure data capture for research purposes. Activity monitor: Fitbit.	Daily steps were assessed by feasibility (defined a priori as a 60% approach-to-consent ratio and 70% Fitbit adherence), acceptance (defined by 20% of participants expressing burden or regret for participation), and preliminary effectiveness (defined by 70% reporting greater motivation). At the end, there was a debriefing interview.	Tracker adherence was 94%. At the 24-week follow-up, 1/24 (5%) of participants reported burden, 0/24 (0%) expressed regret for participating in the study, and 22/24 (>90%) agreed/strongly agreed that “the study pushed me to raise exercise levels”. Participants’ mean daily steps increased from 6210.7 (3328.1) at baseline to 7643 (3610.9) steps (*p* 0.001) during the 12-week intervention. During the 12-week intervention period, participants raised their mean daily steps by 1432 steps and met their step objectives 61.1% of the time. However, twelve weeks after the intervention ended, participants’ mean daily steps dropped to 6435.1 (+3551), which was not significantly higher than their baseline step count (6210 (3328) vs. 6435 (3551), *p* > 0.05). Only 33.9% of individuals met their step objectives during this time period. Working with a teammate was pleasurable for the majority of participants.	6/7
Singh et al. (2020) [66]	Evaluate the effect and acceptability of a PAC session, plus provision of a Fitbit, on maintenance of PA levels 12 weeks following participation in exercise intervention.	RCT. Participants (*n* = 60) were randomized to intervention (*n* = 30) or control (*n* = 30). Baseline and 12- weeks follow up assessments.	Control: PAC. Intervention: PAC + Fibit. Activity Monitor: Self-report questionnaire and Actigraph^®^ GT3X+ accelerometers.	The fulfillment of the following requirements indicates feasibility: 1 Participants wore the Fitbit for at least 10 h per day on at least five out of seven days in a regular week (group mean); 2 More than 80% of participants said the Fitbit was straightforward to use; 3 More than 80% of participants said the Fitbit was comfortable to wear; 4. More than 80% of people were satisfied with the Fitbit as a method of assistance with PA maintenance; 5. Over 80% of participants stated they would keep using the Fitbit in the future.	At 12-week follow-up, the PAC + F group had higher self-reported MVPA and self-reported total activity than the PAC group (between-group mean difference: 78.2 [95% CI = 8.3, 164.9] min/week, *p* 0.01, and 171.9 [95% CI = 46.1, 297.8] min/week, *p* 0.01, respectively). At 12-week follow-up, the PAC + F group had higher objectively-assessed MVPA (*p* = 0.03) and steps/day (*p* = 0.07) than the PAC group. The majority (>80%) of the PAC + F group reported high levels of Fitbit use, indicating the device was useful for PA maintenance. The Fitbit was easy to use (*n* = 24, 92%) and comfortable to wear (*n* = 22, 84%), according to the majority of participants.	5/7
Uhm et al. (2017) [67]	Compare the effects of mobile health (mHealth) and pedometer with conventional exercise program using a brochure on physical function and quality of life.	Randomized clinical trial. Participants (*n* = 356) were randomized to either mHealth with pedometer (*n* = 179) or conventional program (*n* = 177). Baseline and 12- weeks follow up examined the self-reported PA.	Control: exercise brochure. Intervention: home-based prescribed aerobic exercise and resistance exercises + physical therapists + smartphone exercise application (Smart After Care). Activity Monitor: pedometer, and self-reported questionnaire.	Weekly physical activity as indicated by the pedometer user satisfaction questionnaire survey, with responses ranked from 1 (“strongly disagree”) to 5 (“strongly agree”).	Weekly PA increased significantly in both groups, with the increase being greater in the mHealth group but not statistically significant. In the mHealth group, the mean Likert scale response for overall satisfaction with the service was 4.27/5.	2/7
Van Blarigan et al. (2019) [68]	Determine the feasibility and acceptability of the intervention and explore the potential effect of the intervention on accelerometer-measured PA.	Randomized clinical trial. Participants (*n* = 42) were randomized to intervention (*n* = 21) or control (*n* = 21). Baseline and 12- weeks follow up assessments.	Control: usual care. Intervention package: Fitbit Flex™ + daily text message. Activity monitor: Actigraph GTX3+ accelerometers.	Adherence and attrition were used to determine feasibility. Acceptability was measured using a 14-item questionnaire in which participants were asked to rate their level of agreement with four claims regarding text messages and one statement about the Fitbit.	The majority (88%) of the 16 intervention participants who completed the feedback survey reported that the intervention motivated them to exercise and that they were satisfied with their experience. The intervention arm increased their MVPA by 13 min per day more than the control arm (mean difference: 13.1 min per day; 95% confidence interval: −13.5, 39.7). There was no statistically significant difference in MVPA change among arms from baseline to 12 weeks.	2/7
Wang et al. (2011) [69]	Examine the effects of a walking program on Taiwanese women newly diagnosed with early-stage breast cancer.	RCT. Participants (*n* = 72) were randomized to the either exercise group (*n* = 35) or usual care group (*n* = 37). Assessment: (1) 24 h prior to the surgery, (2) 2–3 weeks after surgery, (3) 7–10 days after chemotherapy, (4) the end of the 6-week intervention.	Control: usual care. Intervention package: 6-week walking program + plan to boost exercise self-efficacy (the HR ring monitor + pedometer + weekly phone call + weekly exercise diary + weekly meeting + role model store). Activity monitor: The exercise intensity was measured by HR rings and pedometer.	The retention rate was considered to assess the feasibility. MVPA was defined as a heart rate maximum (HRmax) of 40% to 60% or a modified Borg Scale of 0.5 to 2, 3 to 5 sessions per week, and at least 30 min each session or the accumulation of 10 min sessions to achieve 30 min. Exercise capacity was measured using a 6 min walk distance. During exercise, self-monitoring using the heart rate ring and pedometer. The appropriate exercise intensity was determined using the heart rate.	The retention rate for this study was 86.1%. The exercise group significantly outperformed the usual-care group in terms of exercise behavior following the intervention (F1,60 =13.55, *p* = 0.001).	1/7
Zeng et al. (2020) [70]	Investigates whether a year-long combined fitness wristband-based and personalized exercise prescription intervention improves Chinese breast cancer survivors’ health outcomes.	Pre-post design. baseline (*n* = 95) and post-study (*n* = 33). Baseline and 12-month assessments.	Intervention package: Xiao mi wristband + exercise prescriptionActivity monitor: Xiaomi wristband	In the hospital, health outcomes were assessed. Blood samples were used to measure lipid profile and blood glucose, electrochemiluminescence immunoassay and chemiluminescence enzyme immunoassay were used to examine breast cancer biomarkers, and the Senior Fitness Test (SFT) was used to assess functional fitness. The Xiaomi bracelet measured PA, including steps taken, heart rate, calories expended, and so forth.	The percentage of retention was 35%. Remarkable shifts in functional fitness: agility and balance (MD: −0.47, 95% CI: −0.68–−0.26, t = −4.336, *p* < 0.001), aerobic endurance (MD: 89.25, 95% CI: 73.82–104.68, t = 11.336, *p* < 0.001), lower-body flexibility (left) (MD: 4.58, 95% CI: −4.4–13.56, t = 4.653, *p* < 0.001), and low-er-body flexibility (right) (MD: 4.84, 95% CI: −4.65–14.33, t = 4.092, *p* < 0.001).	4/7
Zhang et al. (2017) [71]	Establish the feasibility and acceptability of completing a higher dose of the planned PA volume among women with ovarian cancer.	Pre-post design. Baseline (*n* = 10); post study (*n* = 10). A total of 10 of 17 were enrolled in a first-contacted, first-served manner. Participants met weekly to download/review weekly PA by step count data.	Intervention package: exercise DVDs + self-reported logs + objective PA monitor (Fitbit). Activity monitor: ActiGraph GT3X triaxial accelerometer.	The adherence rate was used to assess acceptability. Participants were asked for feedback and if they were satisfied with the exercise intervention during the 26-week phone follow-ups. To objectively measure and track adherence to the exercise intervention, participants were requested to wear an activity tracker (Fitbit Zip) for the whole 26-week period. PA is measured as follows: (a) minutes of MVPA (METs), (b) minutes of light-intensity physical activity (1–3 METs), (c) average minutes of MVPA (3 METs or greater), and (d) ambulatory steps using validated cut-points appropriate for adults.	Participants received 83% of in-person sessions with the trainer. The intervention was rated as “very helpful” by all eight research participants. The majority of respondents reported improved function, as evidenced by remarks such as “feeling better” or “more active”. The amount of moderate-intensity movement per day increased by 15 min per day (*p* = 0.05). The number of steps taken per day rose by 1593 (*p* = 0.041). On average, MVPA increased by 10.02 min per day (*p* = 0.078). The amount of time spent on personal activities rose by 15.5 min (*p* = 0.009).	4/7

Abbreviations: applications (apps); body mass index (BMI); confidence interval (CI); cardiovascular disease (CVD); general practitioner (GP); heart rate (HR); mobile health (mHealth); rating of perceived exertion (RPE); type 2 diabetes (T2D); metabolic equivalents (METs); moderate-to-vigorous-intensity physical activity (MVPA); electronic health record (EHR); *p*-value (*p*); physical activity (PA); physical activity counselling (PAC); timepoint(T). ^†^ Reported outcomes and findings are limited to those relevant to wearable activity-monitoring technologies (i.e., impact on activity level, participant motivation, participant adherence to protocol).

## Data Availability

Not applicable.
